# A case report of persistent phrenic nerve injury following pulsed field ablation using a pentaspline catheter

**DOI:** 10.1093/ehjcr/ytag065

**Published:** 2026-01-29

**Authors:** Thet Su Su, Ibrahim Antoun, Riyaz Somani

**Affiliations:** Department of Cardiology, Glenfield Hospital, Groby Road, Leicester LE3 9QP, UK; Department of Cardiology, Glenfield Hospital, Groby Road, Leicester LE3 9QP, UK; Department of Cardiovascular Sciences, University of Leicester, Glenfield Hospital, Leicester LE3 9QP, UK; Department of Cardiovascular Sciences, University of Leicester, Glenfield Hospital, Leicester LE3 9QP, UK

**Keywords:** Atrial fibrillation, Pulsed field ablation, Phrenic nerve injury, Electrophysiology, Case report

## Abstract

**Background:**

Pulsed field ablation (PFA) is an emerging non-thermal ablation modality for atrial fibrillation (AF), characterized by its myocardial selectivity and reduced risk of collateral damage to extracardiac structures such as the oesophagus and phrenic nerve (PN). While transient phrenic nerve injury (PNI) has been reported, persistent diaphragmatic paralysis remains exceedingly rare.

**Case presentation:**

We report the case of a 49-year-old man with paroxysmal AF, refractory to medical therapy, who underwent pulmonary vein isolation using the FARAPULSE PFA system. The procedure was uneventful, and the patient was discharged the same day. However, he reported significant breathlessness on minimal exertion starting the day after the procedure. At his 6-week follow-up, physical examination revealed reduced air entry at the right lung base. A contrast-enhanced computed tomography chest confirmed an elevated right hemidiaphragm (9.0 cm posteriorly, 7.5 cm anteriorly), consistent with right PN palsy. The patient remained in sinus rhythm and asymptomatic from an arrhythmic standpoint, but continues to experience persistent dyspnoea.

**Discussion:**

To our knowledge, this represents one of the early cases of persistent PN palsy following PFA with the FARAPULSE system. Although PFA is designed to target cardiomyocytes while sparing adjacent tissues selectively, proximity to the right PN—particularly near the right superior pulmonary vein—may still pose a risk. This case highlights the importance of procedural vigilance, including meticulous catheter positioning and careful consideration of nerve proximity. It highlights that persistent PNI, while rare, remains a potential complication of PFA.

Learning points

- Persistent phrenic nerve injury is a rare but possible complication of pulsed field ablation, despite its tissue-selective, non-thermal mechanism.
- Precise catheter positioning near the right superior pulmonary vein is critical to minimize the risk of collateral nerve damage.

## Introduction

Atrial fibrillation (AF) stands out as one of the most prevalent arrhythmias, significantly affecting patients’ quality of life while contributing to increased morbidity and mortality rates.^1^ Pulmonary vein isolation (PVI) is an effective treatment for drug-refractory AF. Pulsed field ablation (PFA) is a novel, non-thermal technique that uses high-voltage electrical fields to induce irreversible electroporation of cardiomyocytes, with reported specificity that spares adjacent tissues such as the oesophagus and phrenic nerve (PN). Unlike radiofrequency and cryoballoon ablation, PFA is believed to reduce the risk of collateral tissue damage due to its myocardial selectivity and non-thermal mechanism of action.^2–4^

Whilst transient phrenic nerve injury (PNI) following PFA has been reported, persistent palsy is exceptionally rare. A recent study by Chéhirlian *et al*. demonstrated that electrophysiological compound motor action potential (CMAP)-defined PNI occurs more frequently than previously thought.^5^ In one patient, persistent diaphragmatic dysfunction remained at 3-month follow-up. In this context, we present a detailed clinical case of symptomatic, radiologically confirmed persistent PN palsy following PFA, providing complementary clinical insights.

## Summary figure

**Figure:**
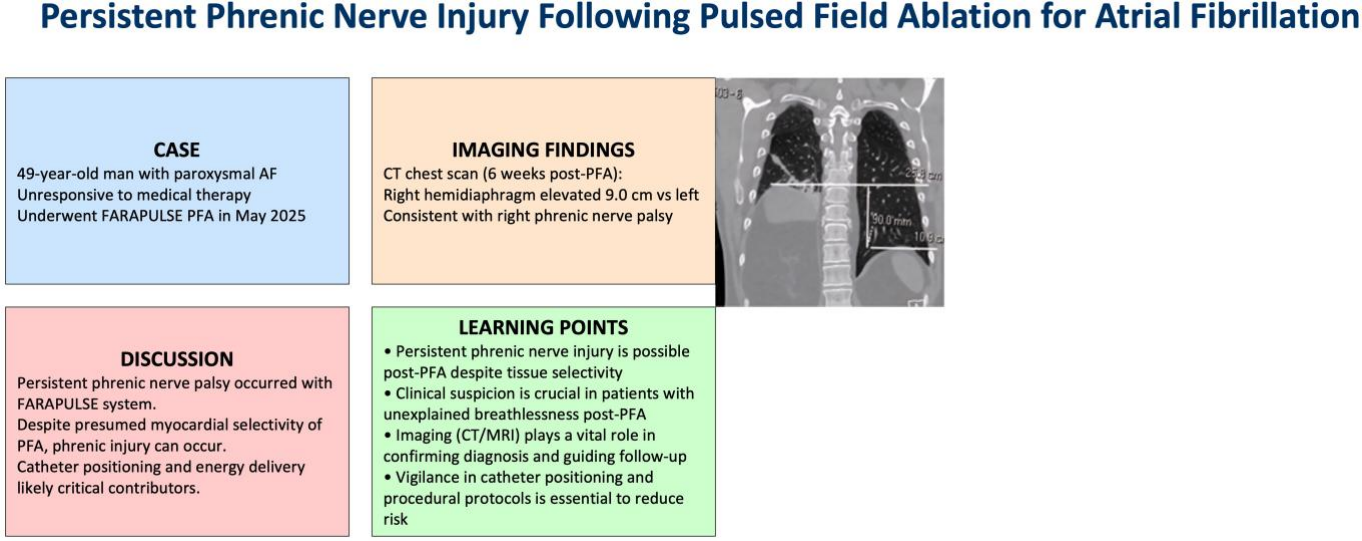


## Case presentation

A 49-year-old male diagnosed with paroxysmal AF 6 years earlier, presented with a 6-month history of palpitations and a significant increase in the frequency and severity of AF episodes, severely affecting his daily life. There was no other significant past medical history.

He was treated medically with a combination of Flecainide 50 mg twice daily and propranolol therapy and anticoagulated with apixaban. Due to ongoing symptoms, PVI was discussed with the patient, who was subsequently admitted electively for the procedure.

A 31 mm pentaspline (Farawave) catheter was introduced through a 13F (Faradrive) deflectable sheath into the left atrium after transseptal puncture. PFA was then carried out sequentially (biphasic, bipolar waveform, microsecond 2.0-kV scale pulses) to all four pulmonary veins (PVs), which were located in their standard anatomical position. A conventional configuration of the pentaspline catheter was used (x4 basket and x4 flower), guided by fluoroscopy with an additional two anteriorly directed applications to the left superior PV and three anteriorly directed applications to the right PV. Care was taken while delivering PFA to the right-sided veins to ensure the catheter remained within the cardiac silhouette, minimizing the risk of inadvertent delivery of ablation within the veins. An additional two anteriorly directed flower applications were delivered in the upper veins, resulting in a total of 37 PV applications. No ablation was conducted outside of the PVs. The patient appeared to have made an uneventful recovery following the procedure and was discharged home the same day.

**Figure 1:**
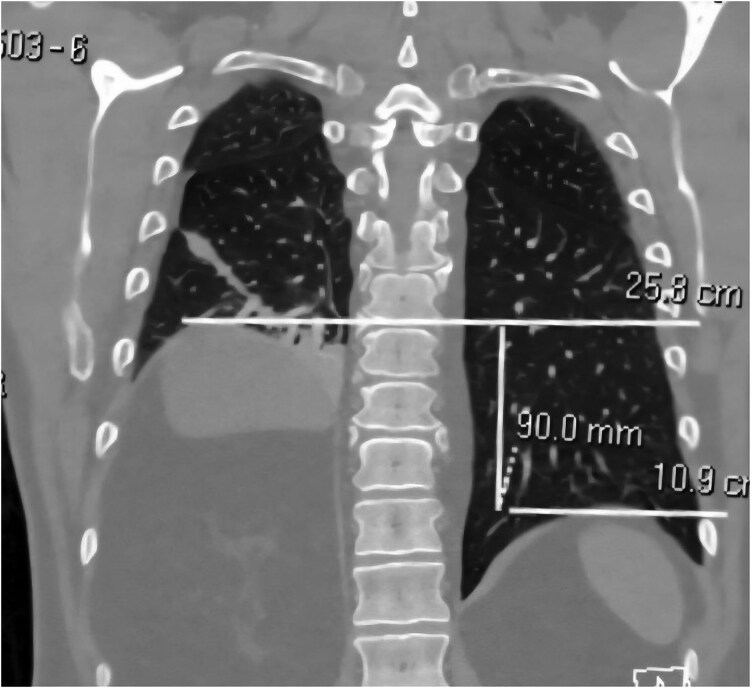
Computed tomography of the chest demonstrating elevated right-sided diaphragm ∼9 cm compared to the left-sided diaphragm.

A follow-up consultation took place 6 weeks post-procedure, earlier than anticipated, prompted by the symptom of breathlessness. The patient reported a significant improvement in his condition. However, he experienced a marked change in his breathing, with substantial breathlessness even on minimal exertion, which he noticed the day after the procedure. Upon examination, reduced air entry was observed at the right lung base, suggesting the possibility of an injury to the right PN. A computed tomography scan of the chest with contrast corroborated this suspicion, revealing an elevated right hemidiaphragm compared to the left side, measuring ∼9 cm at the posterior aspect and 7.5 cm at the anterior aspect (*Figure 1*). The patient was evaluated 2 weeks following the initial clinic visit, which occurred 8 weeks post-procedure. There was no improvement in the symptoms noted at that time, and he was therefore referred to a cardio-respiratory rehabilitation program. The trajectory of his recovery remains uncertain.

## Discussion

This case report represents one of the earliest documented instances of persistent PNI associated with PFA utilizing the pentaspline FARAPULSE system. While transient PNIs are more frequently described, persistent dysfunction remains exceptionally rare. A permanent palsy has previously been noted in the MANIFEST-PF registry, and another was defined using the PulseSelect system, underscoring the need for continued vigilance in monitoring PN integrity during PFA procedures.^6,7^

Furthermore, PFA exhibits a high degree of selectivity towards myocytes, thereby minimizing damage to extra-cardiac cells. As a result, injuries to the oesophagus and PN occur less frequently compared to other ablation methods. In the MANIFEST study, which comprised over 17 000 patients who underwent PFA, there were no reported cases of persistent PNI. Only 11 cases (0.06%) of transient PNI were noted, all of which resolved by the following day.^8^ These findings are consistent with those from the EU-PORIA registry, an observational study conducted across seven European centres, which reported a 0.3% PNI rate with only one persisting at 12-month follow-up.^9^ Subsequent studies, including the FARA-Freedom Study and the Advent Trial, documented no occurrences of PN palsy.^10,11^

Chéhirlian and colleagues reported an unexpectedly high rate of CMAP-detected PNI (40.6%) during PFA procedures. At discharge, incomplete PNI persisted in 24% of their monitored cohort, and in one patient, it continued at 3-month follow-up.^5^ Our case corroborates their findings from a clinical and radiological standpoint. Unlike subclinical electrophysiological changes alone, this case documents overt diaphragmatic dysfunction with symptomatic dyspnoea, highlighting the importance of thorough post-procedural assessment and relevant clinical counselling.

Upon reviewing our case, the aetiology of the PNI remains ambiguous. The placement of the catheter was guided by fluoroscopy, utilizing various views to ensure the basket and flower shapes met the required standards. The energy dose was delivered according to standard practices, using a Farastar generator configured for a standard voltage output and offering a biphasic waveform on a microsecond scale. The prevention and monitoring of PNI remain significant challenges in clinical practice. Unlike the other ablation methods, continuous monitoring of PN activity through PN pacing is not feasible due to the potential movement and instability of the pacing catheter during energy delivery, which could lead to loss of PN capture. Transient PNI during a PFA, as monitored using CMAP, was previously reported to indicate a progressive onset of the palsy, correlating with a dose–effect relationship. This was evidenced by a decrease in CMAP amplitude corresponding to the volume of PFA delivered.^12^

The development of PFA catheters supplemented with anatomical mapping may prove beneficial, as it enables visualization of the catheter and enhances the positioning accuracy. In our case, no intraprocedural abnormalities in diaphragmatic excursion were noted, and no fluoroscopic or CMAP data were stored to evaluate PN function. Unlike cryoballoon or radiofrequency ablation, where PN pacing is routinely performed during ablation of the right PVs, systematic PN monitoring was not part of the PFA workflow at our institution at the time of this case. This represents a limitation of our report. The future integration of dedicated PN monitoring techniques, such as CMAP recording or fluoroscopic tracking of diaphragmatic motion, may be beneficial in detecting subclinical injuries and preventing persistent dysfunction.

Furthermore, the ablation was performed under fluoroscopic guidance without the use of 3D electroanatomical mapping. Although pure fluoroscopic approaches allow for fast and effective procedures, the exact localization of the ablation catheter, relative to extracardiac structures such as the PN, cannot always be accurately judged. This limitation may contribute to inadvertent injury in certain anatomical configurations, supporting the potential role of advanced mapping integration in future practice. Another limitation of this report is the absence of stored fluoroscopic images or catheter position documentation in the right superior PV. Such data would have provided valuable insights into the anatomical proximity of the ablation catheter to the PN.

## Lead author biography


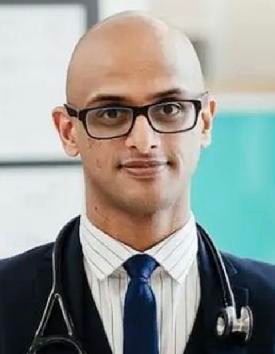


Dr Riyaz Somani is a Consultant Cardiologist and Electrophysiologist who has worked in cardiology since 2002. In December 2014, he was appointed as a Consultant Cardiologist at the Glenfield Hospital, Leicester. He sees private patients at Spire Nottingham and Spire Leicester, treating adults and teenagers aged 16–17. As well as general cardiology, as an internationally trained and highly experienced electrophysiologist, Dr Somani has special expertise in treating palpitations, heart rhythm disorders, and the assessment of sudden cardiac death risk. He performs over 200 procedures per year, which include the following: Pulmonary vein isolation (AF ablation) for paroxysmal and persistent AF and VT ablation with epicardial access.

## Data Availability

Data regarding this case report is available on request from the corresponding author.
